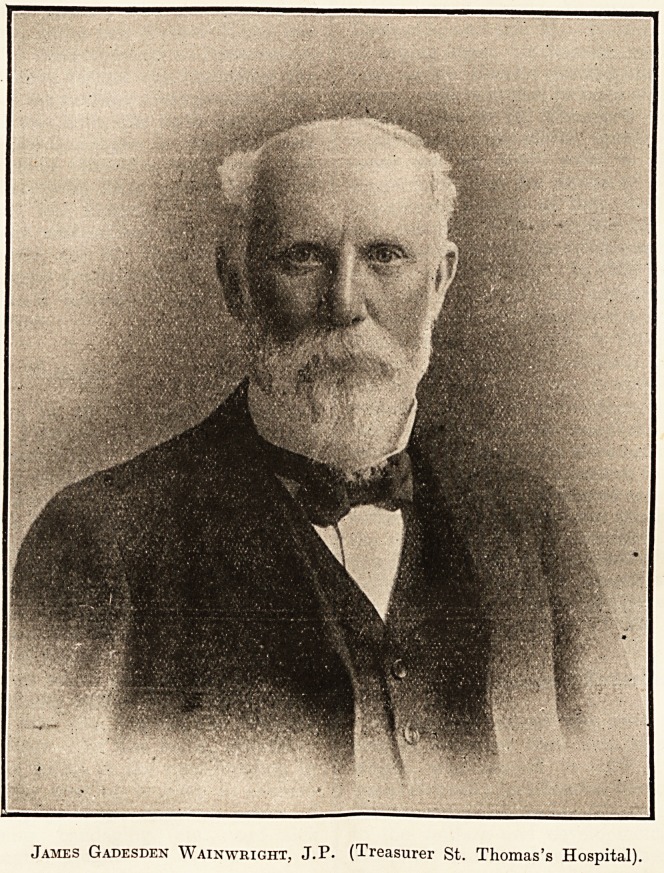# Eminent Chairman Series
*The previous articles in this series appeared in the Hospital of October 1, November 1, and December 10.


**Published:** 1911-01-14

**Authors:** 


### SPECIAL INSTITUTIONAL ARTICLES.

### EMINENT CHAIRMAN SERIES.*

### IV.—MR. JAMES GADESDEN WAINWRIGHT, J.P., Treasurer St. Thomas's Hospital.

What follows is written by one intimately acquainted with the work. St. Thomas's Hospital holds a unique position insomuch as it combines all that is best of old and new. The first definite historical fact we have in regard to it is that the ancient foundation was burned down in 1204. Revenues were set apart for its maintenance when it had been rebuilt by Peter de Roche, Bishop of Winchester, and it was opened as a completely independent foundation in the year 1213. Through succeeding centuries it stood on or near its old site in Bermondsey, endowed with the old lands of Bermondsey Abbey first founded by Edward the Confessor, until it suffered the fate of other religious houses and was surrendered to Henry VIII. To Edward VI. the modern foundation is due, and it is recounted that he, moved by the eloquence of Bishop Ridley, gave back the revenues to the Mayor and Commonalty of London, and appointed it to be called the Hospital of St. Thomas the Apostle, and appropriated it for the poor sick, infirm, and lame persons.

James Gadesden Wainwright, J.P. (Treasurer St. Thomas's Hospital).

The inexorable demands of railway development led to the claim of the site by the London, Brighton, and South Coast Railway, for the Company purchased the site and buildings in 1860, and while a new site was sought, accommodation was temporarily found in the old Surrey Gardens.

The foundation stone of the new building was laid by Queen Victoria in 1868, and the present magnificent buildings were opened for the reception of cases in 1872. At that time the conduct of the Governors in building so large and so complete a hospital was severely criticised, for it was found as soon as the buildings were completed that several wards, capable of containing about two hundred beds, must be left unoccupied, and even unfurnished, owing to lack of funds.

On March 25, 1890, a memorable election to the honorary office of treasurer was held. Fortunately for this great Charity the decision of the Governors was in favour of Mr. James Gadesden Wainwright, for he has devoted himself with indomitable energy to the task of bringing the Hospital to its present state of efficiency when we see, not only all the wards open, but the Charity in a thoroughly sound financial position.

Mr. Wainwright became a Governor of St. Thomas's Hospital in 1866. In 1873 he was elected on the Grand Committee, becoming an Almoner in 1874.

The constitution of St. Thomas's Hospital is a particularly good one. The supreme control of all matters naturally rests with the Governors, but the general government of the hospital is delegated to the Grand Committee—a body of thirty-two Governors elected at the annual Court held in June each year. This Grand Committee meets monthly, while the detailed administration of the hospital is referred to the treasurer and four almoners, meeting weekly, and oftener if necessary at the hospital. The House Committee meets monthly to control

* The previous articles in this series appeared in The Hospital of October 1, November 1, and December 10.

the administration of St. Thomas's Home, and to discuss the general affairs of the hospital, more especially in regard to all matters relating to the medical, surgical, and nursing sides.

In the absence of the President, the Treasurer presides over all Courts of Governors and meetings of the Grand Committee, and as superior officer of the hospital has control over all departments.

The first task undertaken by the present treasurer was the reorganisation of the purchasing departments of the hospital. For instance, the old arrangement of purchasing so many hogsheads of whisky, port, or brandy each year was discontinued, and an account was to be rendered to him giving a detailed explanation of the expenditure. The services of the members of the staff were called in, and with their loyal co-operation and assistance great economies were effected. This system has now been developed until the treasurer is able to keep a very complete check over all expenditure in the wards, not only on drugs and dressings, but on all other purchases of ward necessaries and items of extra diets on which so much money may be unconsciously expended.

Organisation of Finances.

Determined to put the finances on a sound basis, Mr. Wainwright, with the approval and hearty co-operation of the President, H.R.H. the Duke of Connaught, K.G., appealed to the country for help in 1894. Not only did he for the first time publish a report by which the public was informed of the financial position of the hospital, but with the permission of the Lord Mayor, who lent the full weight of his office to furthering so good a cause, a Mansion House meeting was held in February 1895, and a public appeal made for money with the object of enabling the Governors to carry on their work satisfactorily. Many very notable donations were made in answer to this appeal, but none more welcome than the gift of £10,000 by the Mercers' Company for the endowment of beds in a new ward which was opened. The ward received the appropriate name of City Ward, and was devoted to the admirable object of receiving male accident cases. In addition to the twenty beds in the general ward there are two small isolation wards, to which all noisy or particularly serious cases can be taken on their first reception, and special provision is made for immediately dealing with any cases needing operation in the small theatre attached to the ward. Application was made to the Prince of Wales's Hospital Fund on its institution, and on condition that two wards were opened £1,800 per annum was paid as an annual donation to the hospital funds. These wards were accordingly opened, and though they have been maintained continuously, no grant has been made by the King's Fund since 1902.

Ever on the look-out for a means of securing help from his personal friends, Mr. Wainwright approached his old friend, the late Mr. Charles Gassiot, with a view to securing help from him for the building of proper accommodation for nurses. Though a large sum was promised, even the treasurer himself was surprised to find how magnificently Mr. Gassiot had redeemed his promise by making the hospital his residuary legatees. This great bequest has allowed the Governors to meet the heavy expenditure entailed by the complete refitting of the hospital which has been carried on during the last ten years.

The highest praise that can be bestowed upon the able architect, Mr. Henry Currey, who designed

St. Thomas's Hospital, is to state that St. Thomas's was the first great hospital in London to be built on the true pavilion principle, and nothing better has been designed since; in fact, the new King's College Hospital which is being built at Denmark Hill carries out precisely the pavilion system which is the characteristic of St. Thomas's, but in these days of progress it is readily understood that there was much needed to be done in St. Thomas's after thirty years' wear. From this the treasurer has never shrunk, but a new casualty department, new operating theatres, and new children's wards have been built; special accommodation has been provided for the paying patients who are received in St. Thomas's Home, and a new Nurses' Home has been erected. The reorganisation and renovation of the wards has been carried out, including the complete renewal of the sanitary arrangements, for, when this hospital was built, the right and proper thing was to put the soil-pipes in the wall, and now they cannot even be allowed to rest there. The modern method of carrying them outside had to be adopted throughout the Hospital. Provision to meet the present-day medical and surgical requirements has been made, and St. Thomas's is justly proud of the excellent accommodation for *x*-rays, radiant baths, clinical laboratory, and maternity ward for lying-in cases.

### Nursing.

It is well known to all interested in hospital matters that Miss Florence Nightingale honoured St. Thomas's by selecting it as the field for her work in originating trained nursing. When Mr. Wainwright became an active member of the committee he found he had occasion to work closely with Miss Nightingale, and to him, as treasurer, the Matron is responsible for all details of nursing organisation at the present time. Magnificent as the system instituted by Miss Nightingale was, new ideas have arisen, and the treasurer has established a very complete and efficient preliminary training school for nurses, in which all probationers must go through a preliminary trial of six weeks' training before acceptance into the Nightingale Training Home for Probationers. In this home, accommodation for which is found in the new building in Block I., preliminary training is given in elementary anatomy and physiology, and the details of bed-making, nursing, bandaging, etc., and sick cookery are thoroughly taught.

### Medical School.

The close connection between the hospital and the medical school is very fully recognised at St. Thomas's. One great effort of Mr. Wainwright's administration has been to secure the cordial co-operation on all hospital matters of the Governors and the Staff. Not only are representatives of the Staff invited to all meetings of the Governors, Grand and House Committees, where they may, and are invited to, express their opinions on all matters concerning the hospital's welfare, but the Governors have heartily co-operated with the staff in the promotion of the best interests of the medical school, the government of which now rests in a strong committee consisting of five of the Governors, five members of the staff, two co-opted lecturers, and a representative of London University. Mr. Wainwright is not only a member, but takes a very active interest in the deliberations of this body, which has succeeded in bringing the medical school to a high standard of efficiency.

### One Need.

One great need, however, there is at St. Thomas's, and this Mr. Wainwright is never tired of urging upon his friends, seeking a benefactor who, as Mr. Gassiot enabled him to provide for the nurses, will help him to build what is the one thing needed now at St. Thomas's—*a new out-patient department*. Casualties, ophthalmic, and accident cases are all dealt with in the new casualty department; but the rooms of the old out-patient department remain as they were forty years ago, and provision is now needed for the many special departments which the development of medicine and surgery has called for. In 1905 the Treasurer urged upon the Governors the advantages that might be secured by the employment of a lady almoner, whose duties should be not only to check any abuse of the Charity, but organise assistance for patients attending for treatment at the hospital who for various reasons need help to enable them to benefit fully from their attendance at the hospital. In five years this section of the hospital's work has made great strides, and is now serving as a pattern for adoption at other hospitals. Such a record of work ought indeed to suffice for any one man, but this by no means limits the activity of Mr. Wainwright, for he had a distinguished career as chairman of the North Surrey Schools, and is at the present time a member of the committee of the Schiff Home of Recovery, and a most active member of the Central Hospital Council.

### A Memorable Treasurership.

No keener contest was ever conducted for the post of treasurer than that at which Mr. Wainwright was elected in 1890. Mr. Wainwright has abundantly justified the wisdom of his selection. For twenty years he has devoted his whole energies to the welfare and development of St. Thomas's Hospital, and the great benefits conferred upon St. Thomas's through his life-work are here recorded. The voluntary system is entitled to the admiration of the people of all classes throughout the British Empire, but the great and unique practical advantage of this system is strikingly illustrated by attracting to this field of work men of business, of great natural gifts, who take a pride in giving their services and time to the management of our great hospitals. One such man is Mr. Wainwright, who deservedly occupies a high place on the roll of Eminent Hospital Chairmen, and the general hope is that he may long be spared to continue his services to St. Thomas's Hospital.

## Figures and Tables

**Figure f1:**